# Long-Term Whole Grain Wheat and Rye Intake Reflected by Adipose Tissue Alkylresorcinols and Breast Cancer: A Case-Cohort Study

**DOI:** 10.3390/nu11020465

**Published:** 2019-02-22

**Authors:** Huaxing Wu, Cecilie Kyrø, Anne Tjønneland, Katja Boll, Anja Olsen, Kim Overvad, Rikard Landberg

**Affiliations:** 1 Department of Molecular Sciences, Uppsala BioCenter, Swedish University of Agricultural Sciences, SE-75007 Uppsala, Sweden; 2 Danish Cancer Society Research Center, Strandboulevarden 49, DK-2100 Copenhagen, Denmark; ceciliek@cancer.dk (C.K.); annet@cancer.dk (A.T.); katja@cancer.dk (K.B.); anja@cancer.dk (A.O.); 3 Department of Public Health, Section for Epidemiology, Aarhus University, DK-8000 Aarhus, Denmark; ko@ph.au.dk; 4 Department of Biology and Biological Engineering, Food and Nutrition Science, Chalmers University of Technology, SE-41296 Gothenburg, Sweden; rikard.landberg@chalmers.se

**Keywords:** breast cancer, whole grain, rye, wheat, alkylresorcinol, adipose tissue

## Abstract

Whole grain rye (WGR) and whole grain wheat (WGW) have been suggested to protect against the development of breast cancer. In this study, we estimated long-term intake of WGR and WGW, using both a food frequency questionnaire (FFQ) and alkylresorcinol concentrations in adipose tissue biopsies, in relation to the risk of developing invasive breast cancer in a case-cohort study (*n* = 414 in the case group, *n* = 933 in the subcohort group) on the Danish “Diet, Cancer and Health” cohort. The median follow-up time of the subcohort was 5.3 years. Total WGR and WGW intake estimated with FFQ or reflected by total alkylresorcinol concentration in adipose tissue was not significantly associated with risk of breast cancer. However, after adjustment for total WGR and WGW intake, women in the highest quartile of relative WGR intake, reflected by the alkylresorcinol C17:0/C21:0 ratio, had a higher risk of overall breast cancer and estrogen-receptor-positive (ER+) breast cancer than women in the lowest quartile of relative WGR intake, while the risk of estrogen-receptor-negative (ER-) breast cancer incidence was unaffected. Similar results were obtained with the FFQ data. Based on these data, further investigation of the role of specific grain types in reducing or increasing breast cancer risk, and their overall impact on health, is warranted.

## 1. Introduction

Breast cancer is the most frequent cancer in women worldwide, with 1.68 million new cases reported in 2012 [1]. Well-documented risk factors for breast cancer include age, age at menarche and menopause, age at first pregnancy, geographical variation, family history of breast cancer, previous benign breast disease, radiation, the use of oral contraceptive and menopausal hormone, physical activity, alcohol intake, diet, and other lifestyle factors [2]. The etiology of breast cancer is closely related to estrogen receptor (ER) status, with reproduction-related risk factors more consistently associated with estrogen-receptor-positive (ER+) than estrogen-receptor-negative (ER-) breast cancer [3]. 

Whole grains (WG) in the diet have been suggested to reduce the risk of developing breast cancer through the protective effects of dietary fiber and bioactive compounds [4,5,6]. Dietary fiber is proposed to reduce the absorption of mitogenic estrogen and its precursors (cholesterol) in the enterohepatic circulation, thereby lowering the circulating endogenous estrogen level [7,8,9]. Whole grains, particularly rye, are rich in lignans, a group of bioactive compounds with phytoestrogenic activity [10,11,12,13,14]. Human intestinal bacteria metabolize plant lignans to enterodiol and enterolactone, compounds with estrogenic and anti-estrogenic activity that have been associated with a lower risk of breast cancer in some, but not all, observational studies [15,16,17,18]. Plausible mechanisms for this have been suggested, but inconsistent associations between whole grain intake and breast cancer have been reported in observational studies. In one small study with 500 Greek participants, whole grain intake was inversely associated with risk of total breast cancer [19]. Similar results have been reported for premenopausal, but not postmenopausal, US women [20]. In contrast, large prospective studies carried out in Canada [21], US [22,23,24], France [25], UK [26], and Denmark [27] and the European Prospective Investigation into Cancer and Nutrition (EPIC) [28] have shown no significant associations. The inconsistent associations between whole grain intake and breast cancer might be due to most of these studies investigating the role of total whole grain intake, instead of the role of different grains separately, despite the fact that different grains contain different amounts and types of dietary fiber and bioactive compounds [29,30,31,32,33]. For example, rye contains higher amounts of dietary fiber and lignans [34] than wheat [13,35]. In most studies conducted to date, wheat has been the major whole grain source. In addition, whole grain intake in most epidemiological studies has been estimated by self-reporting methods, particularly food frequency questionnaires (FFQ) and dietary recalls [36,37], which are prone to measurement errors. Such measurement errors are inherent to limited databases for whole grain products and to the large variation in whole grain content among whole grain products, intake variation over time, and misreporting by subjects [38]. To overcome these disadvantages, specific dietary biomarkers for objective reflection of whole grain intake are warranted [39].

Alkylresorcinols comprise a group of amphiphilic phenolic lipids mainly present in the bran part of rye and wheat among common foods in the Nordic diet [40,41]. When whole grain rye and/or wheat are consumed, alkylresorcinols with a straight alkyl chain (C15:0, C17:0, C19:0, C21:0, C23:0, and C25:0) [40,41] are absorbed in the small intestine [42], transported in blood [43,44,45], distributed into adipose tissue [46], and eliminated as alkylresorcinol metabolites in urine [47,48,49]. Thus, alkylresorcinol concentration in blood [50,51,52] and adipose tissue [52,53] and alkylresorcinol metabolites in urine [45,54,55] have been evaluated and used as biomarkers of whole grain rye and wheat intake in a number of studies. Moreover, the alkylresorcinol C17:0/C21:0 ratio is around 1.0 in rye and 0.1 in wheat [40,56,57], and, therefore, this ratio in blood is positively associated with relative whole grain rye intake [58]. 

However, the relatively short half-life of alkylresorcinols in blood (~6 h) [58,59] and of alkylresorcinol metabolites in urine (~10 h) [60] limit their application as long-term biomarkers unless the whole grain intake is high and regular [61]. As an alternative, alkylresorcinol concentrations in adipose tissue have been found to be well correlated with long-term whole grain intake from food records in a 12-week whole grain intervention [52] or FFQ [46,53], probably because of their slow turnover. To the best of our knowledge, the association between long-term whole grain rye and wheat intake, reflected by alkylresorcinols in adipose tissue, and the risk of developing breast cancer has not been investigated previously.

The aim of this study was thus to investigate whether total whole grain rye and wheat intake and relative whole grain rye intake (reflected by the ratio of whole grain rye intake and total whole grain rye and wheat intake) are associated with breast cancer risk. Intake was estimated by FFQ or reflected by alkylresorcinols in adipose tissue and the breast cancer risk was assessed for overall, ER+, and ER- cancer, in a case-cohort study in the Danish Diet, Cancer and Health cohort. Our starting hypothesis was that total whole grain rye and wheat intake, and relative rye intake, are inversely associated with risk of breast cancer, especially ER+ breast cancer. 

## 2. Materials and Methods 

### 2.1. Study Population and Breast Cancer Incidence

**Figure 1:**
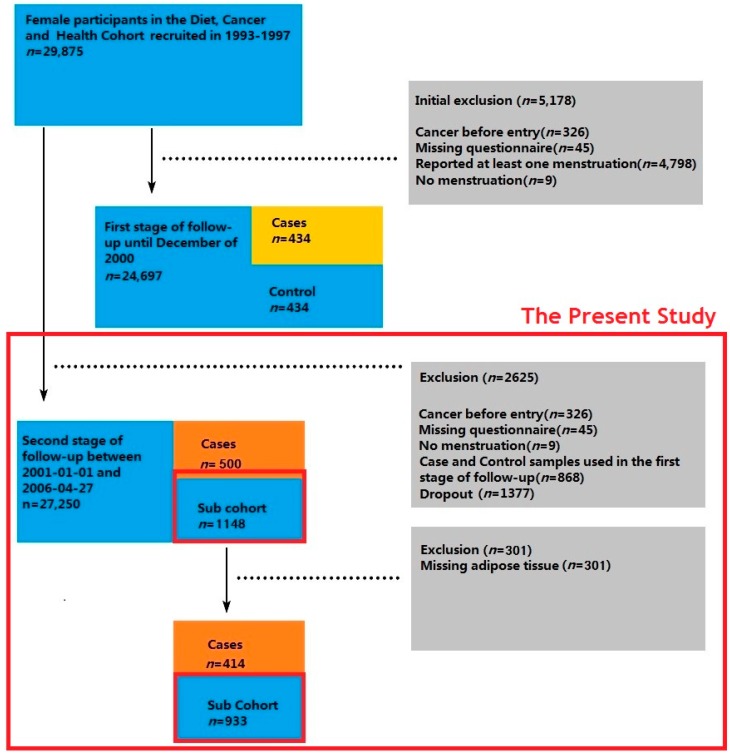
Case-cohort study within the Danish “Diet, Cancer and Health” cohort study between 1993 and chosen-end-of-follow-up (27 April 2006). The first stage of follow-up was between 1993 and December 2000; the second stage of follow-up was between 1 January 2001 and 27 April 2006. The present study is based on the second stage of follow-up.

Women from the prospective Danish “Diet, Cancer and Health” cohort study were included in the study [62] (Figure 1) and the protocol complied with the Helsinki Declaration as revised in 1983. The Diet, Cancer, and Health study was designed to investigate the relations between lifestyle and chronic disease. In total 80,996 men and 79,729 women born in Denmark, living in Copenhagen or the Aarhus area, not registered with a previous diagnosis of cancer in the Danish Cancer Registry, and aged 50–64 at baseline, were invited to participate. The participation among women was 37% and in total 29,875 women were recruited. Subjects were recruited in 1993–1997 (baseline), and all visited one of the two study centers. Prior to visiting the study center, participants completed a 192-item FFQ. At the study center, they completed a lifestyle questionnaire with questions regarding, parity, age at menarche, age at menopause, age at first birth, years of school education, use of menopausal hormones, smoking status, and physical activity. Furthermore, anthropometric measurements were performed, including weight, height, sitting height, waist and hip circumference, and blood samples were drawn by trained personnel. The participants also provided a spot urine sample and toenail clippings. For each participant, 35–50 mg of adipose tissue was taken from the buttock, frozen (−20 °C) within 2 h of collection, and stored in liquid nitrogen vapor (max. −150 °C). Breast cancer cases were identified from the Danish Cancer Registry [63] and the Danish Pathology Register [64]. All procedures performed in studies involving human participants were in accordance with the national ethical standards of the institutional and/or national research committee and with the 1964 Helsinki Declaration and its later amendments or comparable ethical standards. All cohort participants gave written informed consent. The present study was approved by the National Committee on Health Research Ethics (KF 01-345/93) and the Danish Data Protection Agency.

### 2.2. Case Ascertainment and Study Design

Adipose tissue samples for the present study were available from a previous study [65] and, therefore, the same study design, i.e., case-cohort with few modifications, was used (Figure 1). In brief, the women were followed for incidence of breast cancer from baseline until the date of cancer diagnosis, date of death, date of emigration, or 27 April 2006 (chosen end-of-follow-up), whichever came first. Because adipose tissue from the earliest incident breast cancer cases in the first stage of follow-up (diagnosed up to December 2000) was used in a previous study [66], these were not available. This left 713 incident cases and, after retaining only those with invasive ductal or lobular carcinoma and excluding those with rare histology, 574 cases remained. Therefore, the entry date of the present study was changed to 1 January 2001. Breast cancer cases that were diagnosed before 2001 (*n* = 74) were excluded. All invasive breast cancer cases (*n* = 500) were included. A subcohort of non-cases was randomly selected (*n* = 1148) from the cohort, of which 768 were the sub-cohort from a previous case-cohort study [67] and 380 were sampled. After excluding participants with missing adipose tissue samples, 414 breast cancer cases (345 ER+ cases, 56 ER- cases, and 13 ER status unknown cases) and a subcohort of 933 participants (none of whom developed breast cancer during follow-up) remained and were included in the analysis as the second stage of follow-up. 

### 2.3. Dietary Assessment

Diet was assessed from a validated 192-item FFQ described in detail elsewhere [27,68,69,70,71,72]. The participants were asked to report their average intake of food and drink based on the previous 12 months within 12 categories of predefined responses (from “never” to “8 times or more per day”). Intakes of foods and nutrients were estimated from standardized recipes and portion sizes developed using the software program FoodCalc (www.ibt.ku.dk/jesper/foodcalc). Whole grain wheat intake and whole grain rye intake in g/day at baseline were estimated by adjusting the FFQ-based intake of whole grain rye and wheat products (e.g., breakfast cereals, whole grain bread, whole grain crisp bread, whole grain flour and starch, biscuits and crackers, cakes, pies, desserts, and puddings) with their whole grain rye and wheat content reported in a previous study on 3919 randomly selected samples [73] using a standardized 24-h diet record [74], as described in detail in previous studies [71,72]. Estimated total whole grain rye and wheat intake (WGRWGW) was calculated as the sum of FFQ-based whole grain rye intake and whole grain wheat intake. Whole grain rye ratio (WGR%), the ratio of FFQ-based whole grain rye intake to WGRWGW, was calculated to estimate the relative whole grain rye intake. 

### 2.4. Analysis of Alkylresorcinol in Adipose Tissue

Alkylresorcinol concentrations in adipose tissue biopsies were analyzed using a recently developed high throughput GC-MS method [75], with slight modification in the sample clean-up step. In brief, adipose tissue samples (92 samples and quality control adipose tissue samples (*n* = 4) per batch) were defrosted at room temperature, weighed, and mixed with alkylresorcinol internal standard mixture (18.0 ng of alkylresorcinol in 15 μL methanol, which contained equal weight of alkylresorcinol C20:0, alkylresorcinol C22:0, alkylresorcinol C24:0, alkylresorcinol C26:0) and diethyl ether (1 mL) in disposable plastic vials (screw-top microtube, 2 mL, PP, Sarstedt AG, Nürnbrecht, Germany). The plastic vials were loaded with 2.8 mm diameter stainless steel beads (7 per vial), placed in a Retsch MM400 tissue lyser (Retsch GmbH, Haan, Germany), and homogenized at frequency 30 Hz for 2 min. The organic phase from each sample was then transferred to a new disposable plastic vial and dried at 60 °C under nitrogen for 30 min. The extract in the tube was reconstituted in methanol (1 mL) and vortexed for 30 s. The methanol-insoluble fraction of the extract was separated by centrifugation at 14,339 g for 15 min at 4 °C (Eppendorf 5430R, Hamburg, Germany). The supernatant was transferred to a clean test-tube and then applied on a solid phase extraction 96-well plate (Oasis® Max, 60 mg per well, Waters, Milford, MA, USA) as follows: The solid phase extraction plate was conditioned with 1 mL 0.1 M NaOH in methanol (3:7, *v*/*v*); applied with sample, and washed with 3 mL of methanol; alkylresorcinols were eluted with 2% formic acid in methanol (2 mL) and dried under a nitrogen stream at 60 °C. Dried alkylresorcinols were derivatized with 300 μL of trifluoroacetic anhydride (TFAA) at 40 °C for 60 min. Excess TFAA was removed by evaporation at 60 °C for 20 min. TFAA-derivatized alkylresorcinols were reconstituted in 30 μL undecane and analyzed using a Finnigan^TM^ Trace GC Ultra Gas chromatograph coupled to a Finnigan Trace DSQ II mass detector (Thermo Fisher Scientific, Waltham, MA, USA). The derivatized sample or reference standard in undecane solution (2 μL) was injected into a programmed temperature vaporizing injector with a steel liner at 320 °C; alkylresorcinol homologs were separated in a ZB-5MS column (15 m × 0.25 mm × 0.25 μm, Zebron) with helium flow at 1.0 mL/min. The oven temperature was held at 200 °C for 2 min, raised to 310 °C for 2.2 min, and kept at 310 °C for 5 min. The transfer-line and mass detector were held at 310 °C and 250 °C, respectively. Derivatized alkylresorcinols were analyzed under electron impact ionization with selected ion monitoring at *m*/*z* 540 and 316 for C17:0, 568 and 316 for C19:0, 596 and 316 for C21:0, 624 and 316 for C23:0, 652 and 316 for C25:0. Ion *m*/*z* 316 was used for quantification of all target alkylresorcinol homologs and for quantification of internal standard homologs (C20:0, C22:0, and C24:0). Peak area ratio of C17:0/C20:0, C19:0/C20:0, C21:0/C22:0, C23:0/C22:0, and C25:0/C24:0 was calculated for all samples and converted to concentration using a 9-point standard curve constructed from solutions with different concentrations of reference compounds (0.2–29 ng of each alkylresorcinol homolog) and sample mass. All samples were analyzed within 18 batches. Case and non-case samples were randomly selected with a ratio of 1:2 in each batch; laboratory personnel were blinded to the case status. Intra- and inter-batch coefficient of variation (CV) for total alkylresorcinol concentration in the quality control sample was <8% and <10%, respectively.

### 2.5. Statistical Analysis.

Baseline characteristics for the subcohort and the cases are presented as numbers and percentages for categorical variables and as medians with 5th and 95th percentiles for continuous variables. Whole grain rye and wheat intakes levels estimated from FFQ were correlated with alkylresorcinol concentrations in the adipose tissue using Spearman’s rank correlation. 

We applied a case-cohort design as previously described [76] and used a modified Cox Proportional Hazard Model to estimate the association between exposures and breast cancer incidence. Total breast cancer was the main outcome, while ER+ and ER- breast cancer were secondary outcomes. The modified Cox model takes into account the sampled cases using a special weighting system as described by Kalbfleisch and Lawless [77]: Participants in the subcohort participated with person-time in days from 1 January 2001 until censoring, whereas the sampled cases only contributed 0.5 day at the time of diagnosis of breast cancer. Age was used as the time scale in the Cox models. 

Instead of “whole grain rye intake” and “whole grain wheat intake”, “total whole grain rye and wheat intake” and “relative whole grain rye intake” were the exposures of main interest, since they might better reflect the quantity and quality of whole grain-related dietary fiber and bioactive compound intake. “Total whole grain rye and wheat intake” and “relative whole grain rye intake” were estimated from FFQ and used as independent exposure variables: WGRWGW and WGR% (FFQ models), or reflected by total alkylresorcinol concentration and alkylresorcinol C17:0/C21:0 ratio, respectively, in adipose tissue (biomarker models). WGRWGW and WGR% in FFQ models, and total alkylresorcinol concentration and alkylresorcinol C17:0/C21:0 ratio in adipose tissue in biomarker models, were specified continuously with a one-unit increase, representing a 10 g/day increment in WGRWGW and a 10% increase in WGR%, or a 1 nmol/g increment in total alkylresorcinol concentration in adipose tissue and a 0.1 unit increase in alkylresorcinol C17:0/C21:0 ratio in adipose tissue. These exposures were also categorized according to quartiles among all participants and entered into separate models with the lowest quartile as the reference. Hazard Ratio (HR) with 95% confidence intervals (CI) was calculated for:

(1) **Crude FFQ models**: WGRWGW + WGR% + Total energy intake.
(2) **Multivariate-adjusted FFQ models**: Energy-adjusted WGRWGW (the residual method) + WGR% + Total energy intake + Other breast cancer risk factors.
(3) **Crude biomarker models**: Total alkylresorcinol concentration in adipose tissue + Alkylresorcinol C17:0/C21:0 ratio in adipose tissue.
(4) **Multivariate-adjusted biomarker models**: Total alkylresorcinol concentration in adipose tissue + Alkylresorcinol C17:0/C21:0 ratio in adipose tissue + Other breast cancer risk factors.

“Other breast cancer risk factors” included established breast cancer risk factors at baseline, including body mass index (BMI) (<20, 20–25, and >25 kg/m^2^), education (<8, 8–10, and >10 years in school), parity (0–6, entered as continuous variable), age at first birth (<25, 25–29, and >29), age at first period (<12 or ≥12), menopause status at baseline (pre/post), menopausal hormone use (never/former/current), physical activity (<30 or ≥30 min/day), smoking (never/former/current), and alcohol intake (continuous variable).

In a secondary analysis, we also assessed the risk of developing breast cancer using short alkylresorcinol homologs (C17:0, C19:0 or C21:0) as exposure in **Crude biomarker models** and **Multivariate-adjusted biomarker models.** This was because alkylresorcinol homologs shorter than C21:0 have been shown to suppress synthesis of sex hormones [78] and because the C17:0/C21:0 ratio is potentially affected by C17:0, C21:0, or both. 

SAS statistical analysis software (release 9.4; SAS Institute, Cary, NC) was used for all tests and *P* < 0.05 was considered significant. The MEANS and FREQ procedures in SAS were used for descriptive analyses. The CORR procedure was used for correlation analyses and the PHREG procedure was used for the Cox proportional hazard models.

## 3. Results

The baseline characteristics of cases and of the subcohort are presented in Table 1. The median follow-up time of the subcohort was 5.3 years. Estimated whole grain rye and wheat intake, total alkylresorcinol concentration in adipose tissue, age at baseline, waist-hip ratio, BMI, parity, age at first period, age at menopause, age at first birth, years in school, smoking status, and exercise appeared similar for cases and subcohort members, whereas higher reported alcohol intake and alkylresorcinol C17:0/C21:0 ratio and slightly more frequent current use of menopausal hormones were found among cases.

Total alkylresorcinol concentration in adipose tissue was significantly correlated with whole grain wheat intake (*r* = 0.13, *P* < 0.001), whole grain rye intake (*r* = 0.28, *P* < 0.001), and total whole grain rye and wheat intake (*r* = 0.31, *P* < 0.001). Alkylresorcinol C17:0/C21:0 ratio was correlated with WGR% (*r* = 0.16, *P* < 0.001). 

Total whole grain rye and wheat intake, reflected by WGRWGW or total alkylresorcinol concentration in adipose tissue, was not linearly associated with risk of breast cancer (Table 2). Women in the second quartile (Q2) of total alkylresorcinol concentration in adipose tissue had a 138% or 125% higher risk of developing ER- breast cancer than women in Q1 (Table 2) in the crude and multivariable-adjusted models, respectively. 

Women in the fourth quartile (Q4) of WGR% had a 60% higher risk of developing breast cancer, or a 62% higher risk of developing ER+ breast cancer, than women in Q1, while a non-significant dose-response trend was observed across Q1–Q4. However, a consistent, dose-dependent positive association was observed between alkylresorcinol C17:0/C21:0 ratio in adipose tissue and risk of total breast cancer, with 7–9% higher risk of overall breast cancer per 0.1 unit increment in the ratio in the crude and multivariable-adjusted models (Table 2). Alkylresorcinol C17:0/C21:0 ratio in adipose tissue was positively associated with the risk of developing ER+ breast cancer, but not ER- breast cancer, in the crude and multivariable-adjusted models (Table 2). Interestingly, on investigating whether shorter alkylresorcinol homolog concentrations (C17:0–C21:0) were associated with the risk of developing breast cancer, we found that higher concentrations of the shortest alkylresorcinol homolog (C17:0), but not C19:0 and C21:0, were associated with increased risk of developing ER+ breast cancer (HR = 9.64, 95%CI:(1.22–73.39) per 1 nmol/g increment, *P* = 0.03, adjusted for other breast cancer risk factors), but not with any increased risk of developing ER- breast cancer.

## 4. Discussion

In this study, where whole grain rye and wheat intake were assessed using both questionnaire data and a long-term biomarker, no overall association was found between total whole grain rye and wheat intake and the risk of breast cancer. However, sub-analysis revealed that higher alkylresorcinol C17:0/C21:0 ratio, reflecting higher relative whole grain rye intake, was associated with a higher risk of breast cancer, a finding which warrants further investigation.

Our study has several limitations. Firstly, we only had access to samples and data from women who developed breast cancer between 2001 and 2006, which means long latency between single determinations of whole grain intake/alkylresorcinol concentrations in adipose tissue, which occurred at baseline (1993–1997), and the development of breast cancer. Secondly, our biomarkers only reflected the intake of whole grain rye and wheat, and not that of other whole grains such as oats, since alkylresorcinol are not present in oats. Thirdly, the role of potential dietary and non-dietary determinants of alkylresorcinol C17:0/C21:0 ratio in adipose tissue, other than whole grain rye and wheat intake, has not been studied in detail so far and, therefore, our results might be confounded by as yet unknown factors. 

However, our study also has several strengths. Firstly, Nordic adults seem to maintain a rather stable whole grain intake [53], which means that the whole grain intake of women in the present study might not have differed significantly between baseline (1993–1997) and the second stage of follow-up. Secondly, we tried to improve the estimation of long-term whole grain rye and wheat intake by using both FFQ and alkylresorcinol concentrations in adipose tissue, since these two methods are subjected to uncorrelated measurement errors. Thirdly, the study population was relatively large and its whole grain intake was high and diverse. These properties made the current population suitable for investigation of the effects of long-term whole grain rye and wheat intake on the risk of breast cancer. 

Total alkylresorcinol concentrations and C17:0/C21:0 ratio in adipose tissue observed in the present study were similar to those reported previously for Swedish subjects, but somewhat lower than reported for Finnish subjects [46,52,79], values which follow whole grain product intake reported in these countries [33,80]. The correlation coefficient between FFQ-based intake of whole grain rye and/or wheat and total alkylresorcinol concentration in adipose tissue was within the range reported in previous observational studies [46,53], but lower than that reported in a controlled intervention where whole grain intake was estimated with 3×4-day food records [52]. This is likely due to poor precision in the estimation of whole grain intake, especially for whole grain wheat, using FFQ [53,81]. 

Total whole grain rye and wheat intake (WGRWGW, or total alkylresorcinol concentration in adipose tissue) were not associated with the risk of developing breast cancer in the present study. This finding is in agreement with results from a prospective cohort analysis including all women in the Danish Diet, Cancer and Health cohort study [27]. Our results suggest a limited role of total whole grain rye and wheat intake in the prevention of breast cancer incidence in the study population. This is supported by seven out of nine previous studies showing no significant inverse association between total whole grain intake and breast cancer risk [19,20,21,22,23,24,25,27,28]. 

The lack of association between total whole grain rye and wheat intake might also be due to whole grain rye and whole grain wheat being differently associated with the risk of developing breast cancer, which has been difficult to examine in previous studies. In the present study, and in the previous prospective analysis in the same population [27], there was no indication of any significant associations for either whole grain wheat or whole grain rye intake derived from FFQ when investigated separately in relation to breast cancer. However, after adjustment for total whole grain rye and wheat intake in the present study, we found a robust, dose-dependent association between alkylresorcinol C17:0/C21:0 ratio in adipose tissue and total breast cancer risk for ER+ breast cancer, but not for ER- breast cancer. A similar trend was found for relative rye intake (WGR%) in FFQ-based models. Alkylresorcinol C17:0/C21:0 ratio is highly dependent on the relative whole grain rye to total whole grain rye and whole grain wheat intake, as shown in different populations, and has been suggested as a biomarker of relative whole grain rye intake [58,59,79,82,83]. Our results suggest that higher relative whole grain rye intake, reflected by higher alkylresorcinol C17:0/C21:0 ratio in adipose tissue, or other, as yet unknown factors that could increase alkylresorcinol C17:0/C21:0 ratio are associated with increased risk of post-menopause breast cancer. This potentially occurs through a hormone-dependent mechanism on susceptible epithelium [84], since the risk of ER- breast cancer was not affected. This supports findings in a recent study that daily rye intake during adolescence and midlife is positively associated with breast cancer among post-menopause Icelandic women [85]. However, a previous Danish study [27] on the Danish “Diet, Cancer and Health” cohort did not find any sign of an association between whole grain rye intake (only from FFQ) and risk of breast cancer during the first and second stage of follow-up (Figure 1, median age at entry around 55). Since the women in the present study were older (median age at entry around 60), this may indicate that a rye-related and hormone-dependent mechanism has a profound impact in older post-menopausal women, or that post-menopause breast cell carcinogenesis may be induced by cumulative exposure to the long-term rye-related intake of carcinogens. Theoretically, high intake of the short alkylresorcinol homolog (alkylresorcinol C17:0) mainly derived from whole grain rye [40,56,57] will result in a high C17:0/C21:0 ratio. Adipose tissue in the breast is one of the main sites of sex hormone production in post-menopausal women [86,87]. Short alkylresorcinol homolog exposure of adipose tissue through storage in its adipocytes or in postprandial blood [43] may alter sex hormone synthesis by promoting the synthesis of progestogens from cholesterol and suppressing downstream metabolism of progestogens and estradiol, as shown in vitro [78]. A high concentration of these sex hormones is strongly associated with risk of breast cancer [88]. In support of this theory, using multivariable-adjusted models we found that the risk of developing ER+ breast cancer was positively associated only with the concentration of alkylresorcinol C17:0, and not with the concentration of longer alkylresorcinol homologs (C19:0 and C21:0). Future dietary intervention studies in animals or humans are needed to investigate the effect of whole grain rye and alkylresorcinol C17:0 intake on sex hormone synthesis in women and to identify other, as yet unknown, confounding factors that may explain biological mechanisms underlying the association between C17:0/C21:0 ratio and breast cancer found in the present study. 

## 5. Conclusions

We found no evidence of a protective role of high total whole grain rye and wheat intake, estimated by FFQ and reflected by total alkylresorcinol concentration in adipose tissue, against the development of breast cancer in the study population of Danish women. However, we found that a higher alkylresorcinol C17:0/C21:0 ratio in adipose tissue, which partly reflects higher relative whole grain rye to whole grain wheat intake and a high intake of alkylresorcinol C17:0, was associated with a higher incidence of ER+ breast cancer. This observation was confirmed by a similar finding for relative whole grain rye to whole grain wheat intake data from FFQ models. This warrants further studies on the role of different grains, the use of C17:0/C21:0 ratio in adipose tissue as a biomarker in relation to breast cancer risk, and the potential role of the short alkylresorcinol homolog C17:0 in the risk of developing ER+ breast cancer.

## Figures and Tables

**Table 1:** Characteristics at baseline of women in the case and subcohort groups in a case-cohort study on the Danish Diet, Cancer and Health cohort.

Variable	Subcohort (*n* = 933)	Case (*n* = 414)
	Median (P5–P95) or *n* (%)	
Age at study entry (2001/01/01) (year)	60.8 (54.9–69.0)	61.4 (55.0–69.0)
Waist-hip ratio ^1^	0.8 (0.7–0.9)	0.8 (0.7–0.9)
Body mass index (kg/m^2^) ^1^		
<20	52 (6%)	15 (4%)
20–25	453 (49%)	213 (51%)
>25	428 (46%)	186 (45%)
Alcohol intake (g/day) ^1^	8.6 (0.5–40.2)	10.8 (0.6–41.5)
Whole grain intake (g/day) ^1^		
Wheat	3.4 (0.1–13.0)	3.3 (0.1–13.5)
Rye	21.3 (6.4–53.5)	21.3 (6.4–53.3)
Energy intake (kJ/day) ^1^	8583 (5594–12712)	8574 (5641–12426)
Alkylresorcinols in adipose tissue ^1^		
Total alkylresorcinol (nmol/g)	0.90 (0.31–2.11)	0.88 (0.32–1.95)
C17:0/C21:0	0.21 (0.06–0.45)	0.23 (0.08–0.46)
Estrogen receptor		
Positive	-	56 (14%)
Negative	-	345 (83%)
Unknown	-	13 (3%)
Years in school (years) ^1^		
<8.0	308 (33%)	125 (30%)
8.0–10.0	467 (50%)	212 (51%)
>10	158 (17%)	77 (19%)
Parity ^1^		
Nulliparous	124 (13%)	62 (15%)
1	142 (15%)	76 (18%)
2	428 (46%)	185 (45%)
3	192 (21%)	69 (17%)
4	42 (5%)	18 (4%)
≥5	5 (1%)	4 (1%)
Age at first period (years) ^1^		
<12	224 (24%)	102 (25%)
≥12	673 (72%)	297 (72%)
Unknown	36 (4%)	15 (4%)
Age at first birth (years) ^1^		
<25	619 (66%)	255 (62%)
25–29	233 (25%)	130 (31%)
>29	81 (9%)	29 (7%)
Menopause status ^1^		
Post-	781 (84%)	344 (83%)
Pre-	152 (16%)	70 (17%)
Use of menopausal hormones ^1^		
Never	511 (55%)	177 (43%)
Former	145 (16%)	53 (13%)
Current	277 (30%)	183 (44%)
Unknown	0 (0%)	1 (0%)
Exercise (min/day) ^1^		
<30	379 (41%)	162 (39%)
≥30	554 (59%)	252 (61%)
Smoking status ^1^		
Never	407 (44%)	194 (47%)
Former	214 (23%)	92 (22%)
Current	312 (33%)	128 (31%)

^1^ Variables from baseline of the Danish “Diet, Cancer and Health” cohort study (1993–1997), Abbreviations: P5, 5th percentile; P95, 95th percentile.

**Table 2:** Hazard ratio and 95% confidence interval of total, estrogen receptor-positive, and estrogen receptor-negative breast cancer across quartiles of total whole grain rye and wheat intake and relative whole grain rye intake estimated with a food frequency questionnaire and alkylresorcinol concentrations in adipose tissue (biomarker) ^1^. A case-cohort study in the Danish Diet, Cancer and Health cohort.

Exposure	All Breast Cancer		ER+ Breast Cancer		ER- Breast Cancer	
	Crude Model HR (95%CI)	Multivariable- Adjusted Model HR (95%CI) ^1^	Crude Model HR (95%CI)	Multivariable- Adjusted Model HR (95%CI) ^1^	Crude Model HR (95%CI)	Multivariable- Adjusted Model HR (95%CI) ^1^
**FFQ models**						
WGRWGW (g/day)						
Q1	1	1	1	1	1	1
Q2	0.85(0.59–1.22)	0.98(0.68–1.41)	0.77(0.52–1.13)	0.95(0.65–1.39)	1.71(0.68–4.29)	1.24(0.53–2.93)
Q3	0.86(0.62–1.20)	1.10(0.76–1.60)	0.77(0.54–1.09)	0.98(0.66–1.45)	1.70(0.75–3.84)	1.30(0.56–3.00)
Q4	0.98(0.69–1.38)	0.97(0.67–1.40)	0.89(0.62–1.29)	0.93(0.63–1.37)	1.55(0.64–3.75)	1.34(0.57–3.14)
*Per 10 g/day*	*1.00(0.92*–*1.09)*	*1.01(0.91*–*1.11)*	*0.99(0.90*–*1.08)*	*1.01(0.90*–*1.12)*	*1.02(0.88*–*1.19)*	*0.98(0.8*–*1.21)*
WGR%						
Q1	1	1	1	1	1	1
Q2	1.11(0.77–1.60)	1.10(0.77–1.58)	1.17(0.79–1.73)	1.17(0.79–1.71)	0.83(0.33–2.09)	0.86(0.36–2.05)
Q3	1.15(0.78–1.69)	1.18(0.81–1.72)	1.19(0.79–1.80)	1.18(0.78–1.77)	1.03(0.42–2.54)	1.18(0.52–2.66)
Q4	**1.44(1.03** **–2.03)**	**1.60(1.10–2.35)**	**1.50(1.05–2.15)**	**1.62(1.09–2.42)**	1.31(0.59–2.89)	1.48(0.62–3.52)
*Per 10%*	*1.06(0.98*–*1.14)*	*1.06(0.97*–*1.16)*	*1.07(0.98*–*1.16)*	*1.06(0.97*–*1.16)*	*1.06(0.91*–*1.24)*	*1.10(0.91*–*1.34)*
**Biomarker models**						
Total AR (nmol/g)						
Q1	1	1	1	1	1	1
Q2	0.95(0.68–1.32)	1.01(0.71–1.44)	0.78(0.54–1.10)	0.84(0.58–1.22)	**2.38(1.07–5.28)**	**2.25(1.01–5.00)**
Q3	0.80(0.57–1.12)	0.86(0.60–1.24)	**0.69(0.49–0.99)**	0.77(0.52–1.13)	1.53(0.66–3.57)	1.37(0.57–3.29)
Q4	0.89(0.64–1.23)	0.93(0.65–1.32)	0.84(0.59–1.18)	0.91(0.63–1.32)	1.03(0.41–2.56)	0.93(0.38–2.31)
*Per 1 nmol/g*	*0.99(0.81*–*1.21)*	*1.00(0.80*–*1.24)*	*1.02(0.81*–*1.27)*	*1.05(0.82*–*1.33)*	*0.81(0.55*–*1.20)*	*0.75(0.49*–*1.16)*
C17:0/C21:0						
Q1	1	1	1	1	1	1
Q2	1.40(0.99–1.98)	**1.46(1.01–2.09)**	1.38(0.95–1.99)	1.40(0.95–2.07)	1.48(0.65–3.39)	1.54(0.65–3.65)
Q3	**1.63(1.16–2.30)**	**1.65(1.16–2.36)**	**1.61(1.12–2.32)**	**1.60(1.09–2.33)**	2.12(0.97–4.63)	2.25(0.99–5.09)
Q4	**1.87(1.33–2.63)**	**1.96(1.38–2.79)**	**1.95(1.36–2.80)**	**2.01(1.39–2.92)**	1.25(0.53–2.97)	1.29(0.54–3.07)
*Per 0.1*	***1.07(1.03–1.12)***	***1.09(1.04–1.14)***	***1.08(1.03–1.13)***	***1.10(1.04–1.16)***	*1.01(0.95*–*1.08)*	*1.03(0.96*–*1.11)*

^1^ Adjusted for body mass index, education, parity, hormone replacement therapy, age at first birth, age at menopause, age at first period, exercise, smoking status, waist-hip ratio, menopause status, and alcohol intake. Quartile ranges for the sum of FFQ-based total whole grain rye intake and whole grain wheat intake (g/day) are the following: Q1 (0.92–21.84), Q2 (21.84–28.20), Q3 (28.20–38.91), and Q4 (38.91–99.15). Quartile ranges for the ratio of FFQ-based whole grain rye intake to total whole grain rye intake and whole grain wheat intake (%) are the following: Q1 (0.0–75.1), Q2 (75.1–86.4), Q3 (86.4–94.4), and Q4 (94.4–100). Quartile ranges for total alkylresorcinol concentration (nmol/g) are the following: Q1 (0.04–0.61), Q2 (0.61–0.89), Q3 (0.89–1.26), and Q4 (1.26–10.57). Quartile ranges for alkylresorcinol C17:0/C21:0 ratio in adipose tissue are the following: Q1 (0.00–0.15), Q2 (0.15–0.22), Q3 (0.22–0.29), and Q4 (0.29–10.63). Abbreviations: AR, alkylresorcinols; ER+, estrogen receptor-positive; ER-, estrogen receptor-negative; FFQ, food frequency questionnaire; CI, confidence interval; HR, hazard ratio; Q1, first quartile; Q2, second quartile; Q3, third quartile; Q4, fourth quartile; WGRWGW: sum of FFQ-based total whole grain rye intake and whole grain wheat intake. WGR%: ratio of FFQ-based whole grain rye intake to WGRWGW.
